# Propagation-based phase-contrast imaging of the breast: image quality and the effect of X-ray energy and radiation dose

**DOI:** 10.1259/bjr.20221189

**Published:** 2023-09-08

**Authors:** Indusaa Gunaseelan, Alaleh Amin Zadeh, Benedicta Arhatari, Anton Maksimenko, Christopher Hall, Daniel Hausermann, Beena Kumar, Jane Fox, Harry Quiney, Darren Lockie, Sarah Lewis, Patrick Brennan, Timur Gureyev, Seyedamir Tavakoli Taba

**Affiliations:** 1 Discipline of Medical Imaging Sciences, Faculty of Medicine and Health, The University of Sydney, NSW, Camperdown, Australia; 2 School of Physics, Australian National University, ACT, Canberra, Australia; 3 The University of Sydney, Camperdown, New South Wales, Australia; 4 Australian Synchrotron, Australian National Science and Technology Organisation, Clayton, VIC, Australia; 5 Monash Health Pathology Monash Health, Clayton, VIC, Australia; 6 Faculty of Medicine, Nursing and Health Sciences, Monash University, Clayton, VIC, Australia; 7 School of Physics, The University of Melbourne, Parkville, VIC, Australia; 8 Maroondah BreastScreen, Eastern Health, Ringwood, VIC, Australia

## Abstract

**Objectives:**

Propagation-based phase-contrast computed tomography (PB-CT) is a new imaging technique that exploits refractive and absorption properties of X-rays to enhance soft tissue contrast and improve image quality. This study compares image quality of PB-CT and absorption-based CT (AB-CT) for breast imaging while exploring X-ray energy and radiation dose.

**Methods:**

Thirty-nine mastectomy samples were scanned at energy levels of 28-34keV using a flat panel detector at radiation dose levels of 4mGy and 2mGy. Image quality was assessed using signal-to-noise ratio (SNR), contrast-to-noise ratio (CNR), spatial resolution (res) and visibility (vis). Statistical analysis was performed to compare PB-CT images against their corresponding AB-CT images scanned at 32keV and 4mGy.

**Results:**

The PB-CT images at 4mGy, across nearly all energy levels, demonstrated superior image quality than AB-CT images at the same dose. At some energy levels, the 2mGy PB-CT images also showed better image quality in terms of CNR/Res and vis compared to the 4mGy AB-CT images. At both investigated doses, SNR and SNR/res were found to have a statistically significant difference across all energy levels. The difference in vis was statistically significant at some energy levels.

**Conclusion:**

This study demonstrates superior image quality of PB-CT over AB-CT, with X-ray energy playing a crucial role in determining image quality parameters.

**Advances in knowledge:**

Our findings reveal that standard dose PB-CT outperforms standard dose AB-CT across all image quality metrics. Additionally, we demonstrate that low dose PB-CT can produce superior images compared to standard dose AB-CT in terms of CNR/Res and vis.

## Introduction

Breast cancer is the most prevalent cancer affecting females around the world. According to the World Health Organisation, in 2020, 2.3 million females were diagnosed with breast cancer, contributing to 685 000 deaths. Screening programs and diagnostic imaging modalities play a pivotal role in the accurate detection of breast cancer, which is critical in providing timely and effective treatment options in early stages of the disease, resulting in an improved survival rate. Since the introduction of the national breast screening program in Australia in 1991, mortality rates have declined from 74 to less than 50 deaths per 100 000 women aged 50–74, from 2010 onwards.^1^

2D digital mammography (DM), digital breast tomosynthesis (DBT), computed tomography (CT), ultrasound (US) and magnetic resonance imaging (MRI) are clinically relevant modalities currently utilised in breast imaging. It is vital to recognise that each modality plays a unique role in breast imaging and diagnostic assessment, but all are limited by diagnostic efficacy. Currently, DM is recognised as the gold standard modality in breast imaging for screening purposes globally. However, it is well known that the 3D nature of the breast is not fully accommodated in DM’s planar imaging technique due to superimposition of soft tissues. This adversely affects lesion visibility and perceived contrast. DBT is a 3D imaging technique that acquires low dose images at fixed intervals across an arc, adding depth perception to stacked reconstructions. Consequently, lesion localisation is improved, enabling identification of smaller cancers that may be obscured by superimposition in DM.^2^ However, DBT has limited ability to detect microcalcifications, which can be averted by bimodal (DBT and DM) imaging.^3^ Nevertheless, the primary concern associated with the incorporation of DBT within bimodal screening is increased radiation dose to the breast tissue.^4^

US is effective in investigating cysts and suspicions of benign masses. However, US is highly operator dependant and hence, when used in asymptotic patients for screening purposes, it can produce higher rates of false negatives, decreasing specificity.^5^ MRI is the most sensitive breast imaging modality available and can provide both anatomical and functional information about breast cancers and their vasculature. However, specificity is low with high false-positive rates.^6^ Furthermore, MRI is expensive and time-consuming for routine breast screening and when utilised for diagnostic purposes, it has a plethora of contraindications that can limit accessibility. CT is a multiplanar imaging modality that captures the 3D structure of the breast without breast compression. It overcomes limitations imposed by the superimposition of breast parenchyma and improves diagnostic efficacy. Consequently, superior lesion localisation and contrast resolution allows for visualisation of masses and lesions with appropriate anatomical reconstructions to cater for diagnostic assessment, staging and treatment planning.^7^ However, breast CT has limited ability to characterise calcifications, hindered by its lower spatial resolution compared to DM and DBT.^8^ The detection of microcalcifications by CT, although sufficient, remains inferior to DM.^8,9^

All conventional X-ray imaging modalities, including DM, DBT and CT, only utilise differences in absorption of various tissues to produce image contrast. Phase-contrast imaging is a novel technique that exploits both absorption and refractive properties of X-rays for soft tissue differentiation. By measuring distortions to the X-ray beam known as phase shifts, as it travels through a material, further contrast information can be utilised to enhance soft tissue differentiation of weakly attenuating structures. The complex X-ray refractive index of a material can be expressed as $n\left(E\right)=1-\left(E\right)+i\left(E\right)$ , where δ describes the decrement of the real part of the refractive index and will determine the phase shift, β determines attenuation and E is X-ray energy. In comparison, the values of δ can be up to 10^3^ times greater than in soft tissues with weak attenuation when imaged with hard X-rays with energies *E* around 30 keV.^10^ Hence, X-ray phase shift is highly sensitive to minute changes in structural composition and can potentially produce greater contrast amongst soft tissues of similar density than absorption alone.^11^

Phase shifts form the basis of several methods including propagation-based (PB), analyser-based and crystal interferometer-based techniques. PB technique is reliant upon a large sample to detector distance and spatially coherent X-rays; however it is considered to be the simplest technique for implementation.^12^ Preliminary incorporation of PB radiography within the realm of CT has demonstrated superior image quality, with increased contrast and lesion detectability than absorption-based CT (AB-CT).^13^ Research into optimisation of image quality for PB-CT has provided promising results for translation into clinical practice, with adequate tissue differentiation at lower radiation doses compared to AB-CT, facilitating progression into clinical application.^14,15^

X-ray energy has been recognised as an important parameter for image quality in PB-CT. Baran et al^12^ and Tavakoli Taba et al^16^ investigated X-ray energies between 32 and 38 keV and concluded that 32 keV produces the highest quality images in that range. These studies were limited in their ability to provide insight into the effect of X-ray energies that are lower than 32 keV on image quality in samples of similar size. However, Oliva et al^15^ utilised contrast-to-noise ratio (CNR) as the prime indicator of image quality to suggest that an X-ray energy of 28keV leads to maximum CNR. It has also been proposed that variations in the results between studies can be attributed to other factors such as dose, detector used, breast density and experimental setup.^17^ Therefore, the present study investigates a variety of X-ray energies ranging from as low as 28keV to 34keV at doses of 2mGy and 4mGy and measures image quality as a factor of multiple objective metrics, including signal-to-noise ratio (SNR), CNR, visibility, and spatial resolution.

Lim et al^17^ and Wan et al^18^ have previously investigated the relationship between X-ray energies and image quality in PB-CT. While Wan et al^18^ employed a human observer evaluation approach to image quality assessment and concluded that 28-34keV provided superior image quality in PB-CT in comparison to AB-CT, Lim et al^17^ utilised a collective approach incorporating both subjective and objective image quality assessments, suggesting that 30-34keV provided optimum image quality in PB-CT. The present study builds upon the methodical approach of Lim et al^17^ and focuses on objective image quality assessments, while also incorporating evaluations of PB-CT images obtained using a different detector at standard and low radiation doses. By doing so, our study aims to narrow existing research gaps and provide more insights into the relationship between X-ray energy, dose, and image quality in PB-CT.

## Methods

### Mastectomy samples

Thirty-nine mastectomy samples were retrieved post-surgery from patients undergoing mastectomy and scanned within a few hours of excision at the Imaging and Medical Beamline (IMBL) of the Australian Synchrotron (operated by ANSTO). All participants had underlying malignancy or were undergoing mastectomy for risk reduction purposes and provided written consent prior to surgery to permit the use of samples for research purposes. The Human Research Ethics Committee approval (project number: CF15/3138-2015001340) permits retrieval of mastectomy samples from the tissue bank and irradiation of these samples for research purposes.

In this study, mastectomy samples were imaged in 11 cm thin low-absorbing containers without compression, added preservatives and the nipple was consistently positioned at the superior aspect of the container during the PB-CT scans. Any samples that were too large or too small to fit within the 11 cm containers were excluded from the study.

### Experimental Set-up

The IMBL uses a super-conducting wiggler and bent double crystal monochromator system to produce a parallel monochromatic X-ray beam of up to 500 x 940mm in cross-section in the energy range of 20-120keV, with the energy resolution of ΔE/*E* = 10^−3^. A Teledyne-Dalsa Xineos-3030HR flat panel detector with an active area of 296 × 296mm (2988 × 2988 pixels field of view), a pixel pitch of 99 µm and a frame rate of 40fps was used for all the scans. The PB-CT scans were obtained at an object-to-detector distance of 6m and a source-to-detector distance of 143m. All PB-CT images were subsequently reconstructed in the coronal plane at a standard (4mGy) and reduced (2mGy) doses using the filtered back projection method. Image processing algorithms based on the Homogeneous Transport of Intensity equation (TIE-Hom) were applied for phase retrieval as part of the PB-CT reconstructions.^19^ All samples were also scanned using AB-CT for comparative purposes. The imaging conditions for AB-CT scans were similar to PB-CT scans, except that a small object-to-detector distance of 0.19 m was used during imaging and phase retrieval was not applied in the reconstruction. Monochromatic planar X-ray beam at *E* = 32 keV and a standard mean glandular (MGD) dose of 4 mGy was used in all AB-CT scans. To make sure that all image quality differences in the images were due only to objective physical differences, all AB-CT and PB-CT image reconstructions were stored as 32-bit tiff files without any further manipulation or typical greyscale adjustments, which are commonly used in radiography.

### Image quality assessment

A focused objective analysis of overall image quality in AB-CT and PB-CT was conducted using the X-TRACT software (Version 21 Oct 2020) by measuring SNR, CNR, visibility and spatial resolution.^20^ SNR is a function of signal intensity and inherent background noise and is defined by the ratio of average pixel value to standard deviation of the same region. Hence, SNR can be calculated as: $SNR=\frac{\overset{-}{I}}{\sigma },$ where $\overset{-}{I}$ is mean pixel value and σ is standard deviation and is representative of noise. The relationship between SNR and spatial resolution (res) is inversely proportional in conventional X-ray imaging.^21^ To account for this, image quality is normally analysed with SNR and res as a single unit by dividing SNR by res. Using the X-TRACT software, a homogenous square region of interest (ROI) over fatty tissue measuring at least 1 cm^2^ was selected in all images to measure SNR and SNR/resolution (SNR/res). The X-TRACT software derives the point spread function (PSF) of the detector by analysing the power spectrum of image noise, in order to calculate the spatial resolution.^22^While this method does not account for the X-ray source size, this factor has a relatively minor impact on spatial resolution in the experimental setup used in this study, due to the implicit geometrical demagnification factor.^23^

CNR compares the inherent contrast between a feature in the image and the background and is defined by the ratio of differences in signal intensities between structures to standard deviation of the background. The calculation of CNR is represented by: $CNR=\frac{I_{T}-I_{B}}{2\sigma },$ where $I_{T}$ is the signal intensity of the fibro-glandular tissue, $I_{B}$ is the signal intensity of the background (adipose tissue) and σ is standard deviation of the background, representative of the magnitude of noise. CNR as well as CNR/res were calculated by selecting a heterogeneous rectangular ROI consisting of both glandular and fatty tissue.^21^

Visibility measures the ability to differentiate between objects by comparing the intensity of fibro-glandular and adipose tissues in the breast. Visibility (vis) is defined by: $Visibility=\frac{I_{T}-I_{B}}{2\overset{-}{I}},$ and hence CNR=SNRVisibility . Images with superior visibility will possess the ability to distinguish between adipose and fibro-glandular tissues with ease. Data were collected from at least three slices for each sample: one from the top, one from the bottom and at least one slice from the middle of the sample at almost equal distances. Some samples had a large number of slices, in which case a greater number of slices between the selected top and bottom slice were analysed at regular intervals. Data from each slice were used as a separate entry in statistical analysis. Each measure in PB-CT images was compared against the corresponding reference AB-CT image scanned at 32keV and 4mGy to calculate differences in SNR (dSNR), SNR/Res (dSNR/Res), CNR (dCNR), CNR/Res (dCNR/Res) and visibility (dVis).

### Data analysis

Data analysis was conducted on SPSS (Version 28.0.1.1 (14)) using t-test to compare the overall image quality of PB-CT and AB-CT images and using one-way analysis of variance (ANOVA) to examine the effect of X-ray energy on PB-CT image quality at each radiation dose level. Since non-homogeneous variances were observed in the data, Welch’s t-test and ANOVA with Welch’s test were performed in this study. Post-hoc analysis using Games-Howell test was conducted to identify where significant differences exist within the various energy levels.

## Results

**Figure 1.**
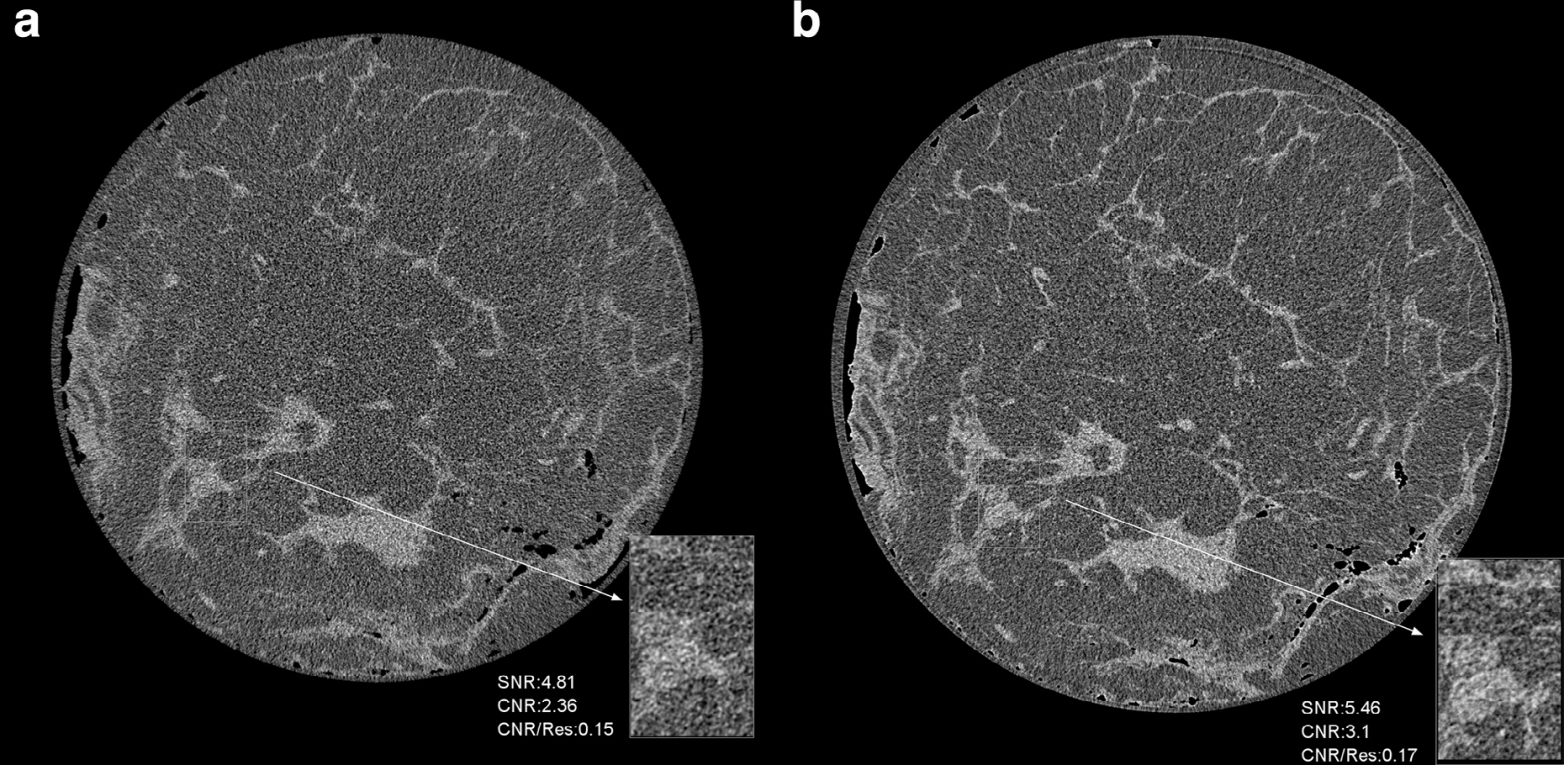
Comparison of Image Quality between PB-CT and AB-CT at the same radiation dose. (**a**) Image obtained with AB-CT at 32 keV and 4mGy. (**b**) Image obtained with PB-CT at 32 keV and 4 mGy, which demonstrates superior image quality. It should be noted that the PB-CT image exhibits a geometrical magnification factor of 1.04 due to the mastectomy sample being scanned at a 6 m object-to-detector distance, compared to the 0.19 m distance in the AB-CT image.

At the standard dose of 4mGy, SNR mean values in PB-CT images were significantly superior to AB-CT reference values at investigated X-ray energies of 32 keV or greater. A similar trend was evident for SNR/res, CNR and CNR/res mean values across all investigated energy levels. Additionally, the mean visibility values were observed to be significantly superior to AB-CT reference value at 28 keV and 30 keV. For 32 keV PB-CT images, visibility was a bit higher compared to AB-CT images, but the difference was not statistically significant. Figure 1 demonstrates comparison between image quality in PB-CT and AB-CT at 32 keV and 4 mGy, with the PB-CT image demonstrating visibly greater image quality. Overall, PB-CT images obtained at 4 mGy demonstrated superior image quality to AB-CT images at 4 mGy across investigated energies.

As evident in Tables 1 and 2, SNR in low energy PB-CT images obtained at 2 mGy were inferior to AB-CT reference images at 4 mGy with demonstrated statistical significance. A similar trend was established with CNR mean values; however, all energy levels except 34keV were statistically significant. Table 3 demonstrates the average spatial resolution as a factor of pixel size for various imaging conditions. The results show that mean values for visibility and CNR/res at all investigated energies as well as SNR/res at 34 keV in PB-CT were superior to AB-CT reference values.

**Table 1.** Mean, standard deviation and *p*-values from Welch’s t-test for measured image quality parameters at 4 mGy. Highlighted cells indicate reference values for AB-CT scans at 32 keV and 4 mGy

Image Quality Metric		Mean	SD	T-test *p*-value
SNR	AB-CT 32 keV Reference	5.73	0.77	
	PB-CT 28 keV	5.28	0.70	<0.001
	PB-CT 30 keV	5.74	0.82	0.905
	PB-CT 32 keV	6.11	0.71	<0.001
	PB-CT 34 keV	7.33	0.88	<0.001
SNR/res	AB-CT 32 keV Reference	2.71	0.34	
	PB-CT 28 keV	3.02	0.38	<0.001
	PB-CT 30 keV	3.22	0.44	<0.001
	PB-CT 32 keV	3.38	0.39	<0.001
	PB-CT 34 keV	4.02	0.48	<0.001
Vis	AB-CT 32 keV Reference	0.11	0.03	
	PB-CT 28 keV	0.14	0.04	<0.001
	PB-CT 30 keV	0.13	0.04	<0.001
	PB-CT 32 keV	0.12	0.03	0.128
	PB-CT 34 keV	0.11	0.03	0.048
CNR	AB-CT 32 keV Reference	0.51	0.19	
	PB-CT 28 keV	0.56	0.21	0.014
	PB-CT 30 keV	0.57	0.23	0.016
	PB-CT 32 keV	0.55	0.22	0.053
	PB-CT 34 keV	0.54	0.22	0.008
CNR/res	AB-CT 32 keV Reference	0.22	0.09	
	PB-CT 28 keV	0.31	0.13	<0.001
	PB-CT 30 keV	0.30	0.13	<0.001
	PB-CT 32 keV	0.29	0.13	<0.001
	PB-CT 34 keV	0.30	0.14	<0.001

**Table 2.** Mean, standard deviation and *p*-values from Welch’s t-test for measured image quality parameters at 2mGy. Highlighted cells indicate reference values for AB-CT scans at 32 keV and 4 mGy

Image Quality Metric		Mean	SD	T-test *p*-value
SNR	AB-CT 32 keV Reference	5.73	0.77	
	PB-CT 28 keV	3.23	0.54	<0.001
	PB-CT 30 keV	3.77	0.62	<0.001
	PB-CT 32 keV	4.02	0.55	<0.001
	PB-CT 34 keV	4.88	0.64	<0.001
SNR/res	AB-CT 32 keV Reference	2.71	0.34	
	PB-CT 28 keV	1.95	0.30	<0.001
	PB-CT 30 keV	2.23	0.33	<0.001
	PB-CT 32 keV	2.35	0.29	<0.001
	PB-CT 34 keV	2.83	0.35	0.001
Vis	AB-CT 32 keV Reference	0.11	0.03	
	PB-CT 28 keV	0.16	0.04	<0.001
	PB-CT 30 keV	0.14	0.04	<0.001
	PB-CT 32 keV	0.13	0.03	<0.001
	PB-CT 34 keV	0.12	0.03	0.134
CNR	AB-CT 32 keV Reference	0.51	0.19	
	PB-CT 28 keV	0.45	0.14	0.002
	PB-CT 30 keV	0.47	0.15	0.026
	PB-CT 32 keV	0.47	0.15	0.023
	PB-CT 34 keV	0.48	0.17	0.105
CNR/res	AB-CT 32 keV Reference	0.22	0.09	
	PB-CT 28 keV	0.26	0.09	<0.001
	PB-CT 30 keV	0.27	0.09	<0.001
	PB-CT 32 keV	0.26	0.09	<0.001
	PB-CT 34 keV	0.27	0.10	<0.001

**Table 3.** Average spatial resolution measured as a factor of pixel size for each image acquisition

Image Acquisition	Resolution
AB-CT Reference	2.13
2 mGy PB-CT 28 keV	1.65
2 mGy PB-CT 30 keV	1.69
2 mGy PB-CT 32 keV	1.71
2 mGy PB-CT 34 keV	1.72
4 mGy PB-CT 28 keV	1.75
4 mGy PB-CT 30 keV	1.78
4 mGy PB-CT 32 keV	1.78
4 mGy PB-CT 34 keV	1.83

Table 4 depicts results from one-way ANOVA with Welch’s test, which indicated a statistically significant difference between X-ray energy levels for measures of dSNR, dSNR/res and dVis at both 4 mGy and 2 mGy. At 4 mGy, energy levels had a significant main effect on dSNR/res, F (3, 366.97) = 295.92 and to a lesser extent on dSNR, F (3, 362.67) = 276.15. A moderate effect of X-ray energy was apparent on dVis, F (3, 375.28) = 34.45. At 2 mGy, larger effects of X-ray energy were apparent on dSNR/res, F(3, 371.19) = 574.96, dSNR, F (3, 370.35) = 493.78 and dVis, F (3, 371.75) = 48.66. Overall, measures of dCNR and dCNR/res were not statistically different at the investigated energies.

**Table 4.** F-statistic and statistical significance values from ANOVA with Welch’s test using calculated differences between PB-CT and AB-CT values for each image quality metric

Image Quality Metric	4mGy		2mGy	
	Statistic	Sig	Statistic	Sig
dSNR	276.150	<0.001	493.677	<0.001
dSNR/Res	295.916	<0.001	574.960	<0.001
dVis	34.445	<0.001	48.664	<0.001
dCNR	1.292	0.277	0.946	0.419
dCNR/res	0.879	0.452	2.279	0.079

Further post-hoc analysis with Games-Howell test in Table 5, revealed that SNR, SNR/res, and Vis were statistically different across all energies at both doses.

**Table 5.** Summary of *p*-values retrieved from post-hoc analysis with Games Howell’s test at investigated X-ray energies

Dose	X-ray Energy		SNR	SNR/res	Visibility	CNR	CNR/res
4 mGy	28	30	<.001	<.001	0.024	0.993	0.982
		32	<.001	<.001	<.001	0.927	0.400
		34	<.001	<.001	<.001	0.472	0.972
	30	32	<.001	<.001	0.002	0.835	0.662
		34	<.001	<.001	<.001	0.674	1.000
	32	34	<.001	<.001	0.004	0.219	0.727
2 mGy	28	30	<.001	<.001	0.006	0.815	0.057
		32	<.001	<.001	<.001	0.852	0.096
		34	<.001	<.001	<.001	0.333	0.057
	30	32	<.001	<.001	0.007	1.000	0.931
		34	<.001	<.001	<.001	0.853	1.000
	32	34	<.001	<.001	<.001	0.809	0.928

**Figure 2.**
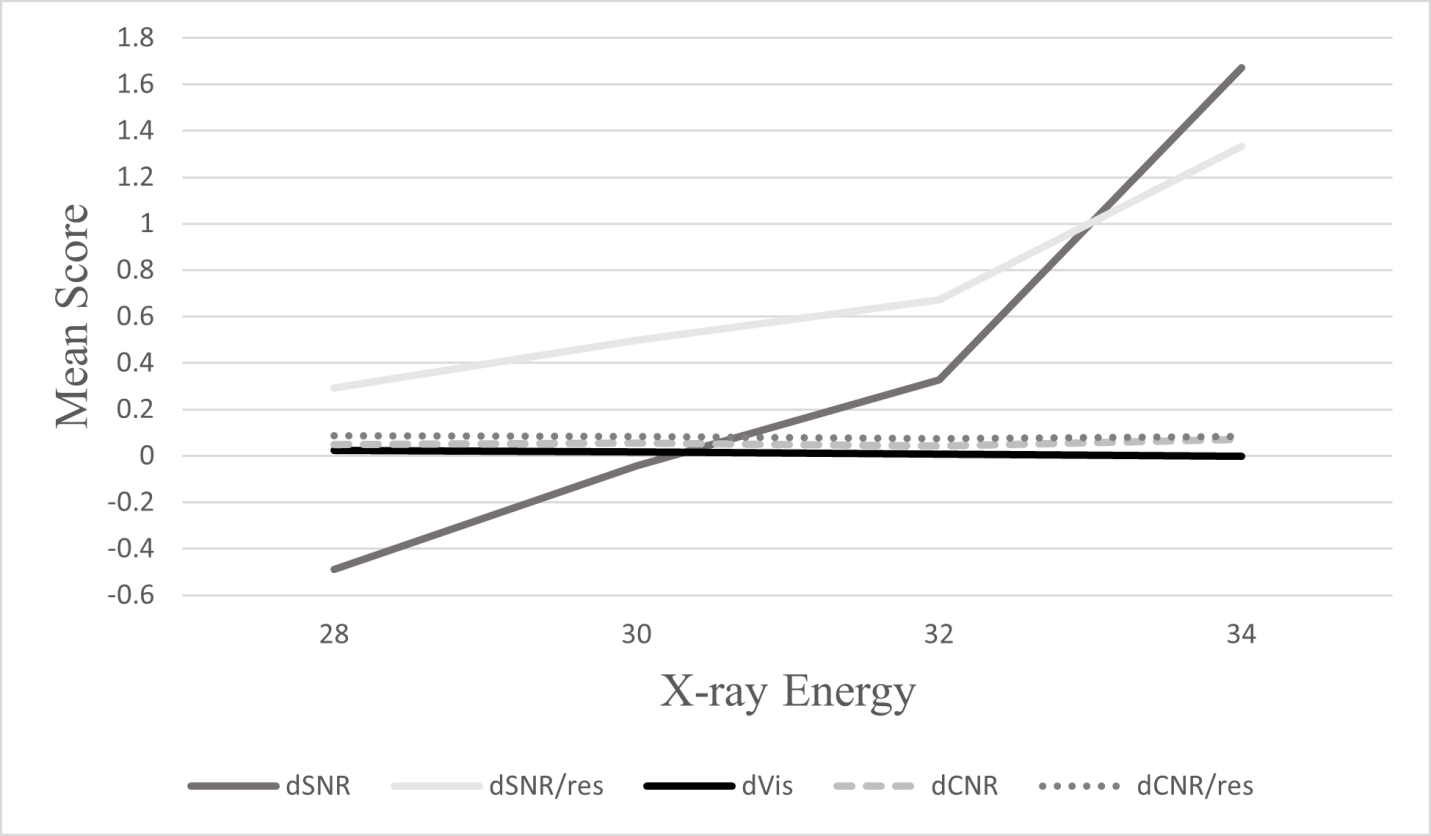
Mean score values for each image quality metric across investigated energies using calculated differences between PB-CT (4 mGy) and AB-CT (4 mGy) values.

**Figure 3.**
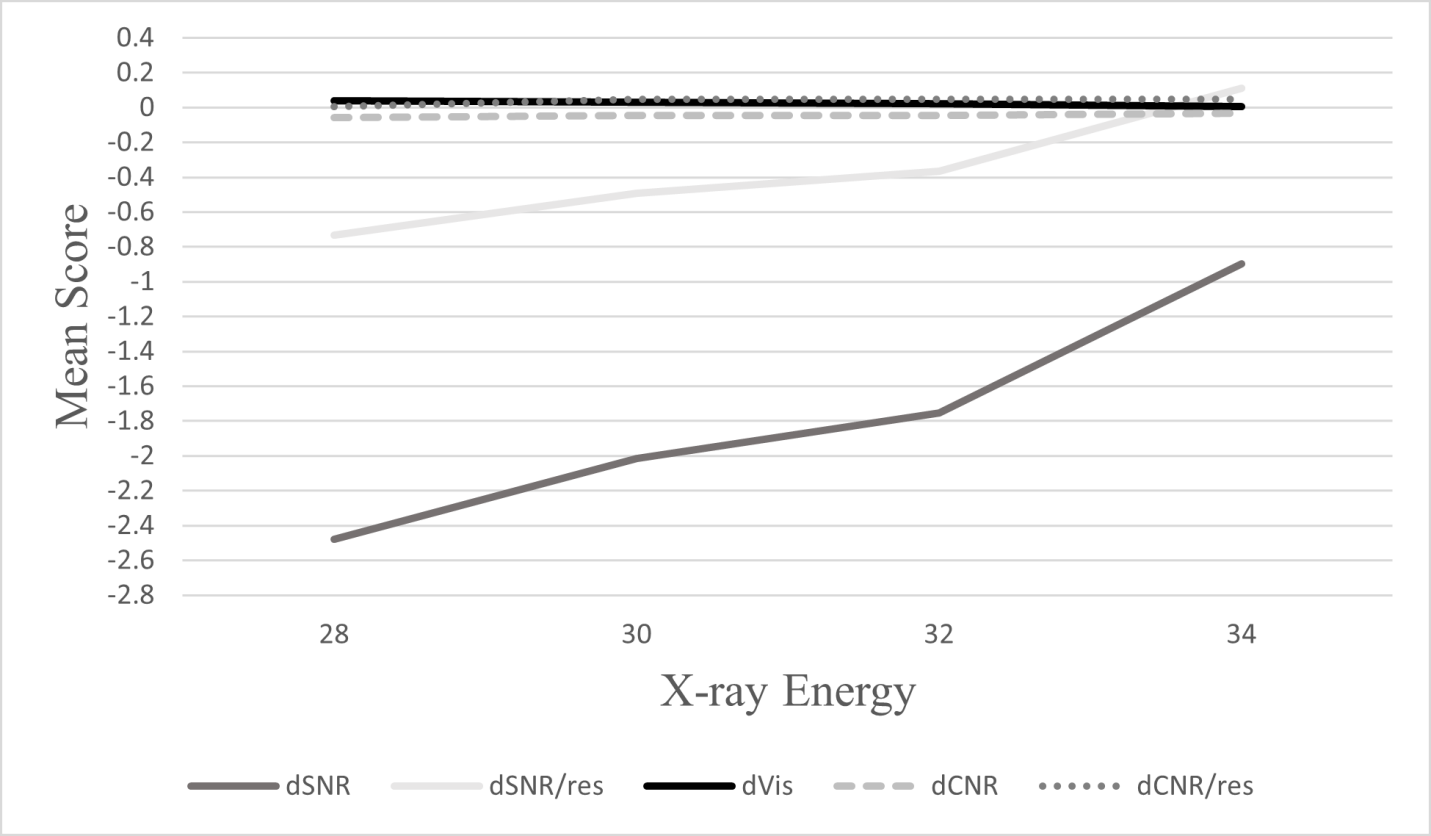
Mean score values for each image quality metric across investigated energies using calculated differences between PB-CT (2 mGy) and AB-CT (4 mGy) values.

As demonstrated in Figures 2 and 3, at both doses, SNR and SNR/res mean scores were increasing with increasing energy and the peak energy level was 34 keV. Minimal changes were evident in mean scores of CNR, CNR/res with increasing energy levels. A slight decline was observed in visibility with increasing energy levels across both doses.

## Discussion

The findings from this study indicate superior image quality in PB-CT imaging compared to AB-CT imaging at standard (4 mGy) doses. It is also shown that X-ray energy is an important factor that influences SNR and SNR/res significantly and to a lesser degree visibility in PB-CT. Similar to conventional X-ray imaging, higher X-ray energies resulted in higher SNR and SNR/res at both standard and reduced radiation doses. At higher energies, there is an increase in the number of photons that reach the detector at the same dose, resulting in greater signal transmission to improve SNR and SNR/res. This is further emphasised by the post-hoc analysis which revealed that SNR and SNR/res were statistically different across all investigated energies at both doses. On the contrary, visibility reduced with increasing X-ray energy, with 28 keV providing the highest mean values across both doses. CNR and CNR/res were not found to be statistically significant across investigated energy levels, indicating that X-ray energy has little effect on CNR and CNR/res values. This can also be related to the fact that CNR is a function of Vis and SNR, so the effects are neutralised.

F-statistic values from one-way ANOVA were significantly greater at 2 mGy compared to 4 mGy images, indicating that at lower doses there is an increased dependence on X-ray energy across all investigated image quality metrics.

The findings from this study are predominantly consistent with a previous study conducted by Lim et al,^17^ demonstrating similar findings in correlation to SNR, SNR/res and visibility. Both studies use a flat panel detector that are similar in composition, however this study employs the new Teledyne-Dalsa Xineos-3030HR detector consisting of a faster frame rate than the Hamamatsu C10900D detector used in Lim’s study. However, Lim et al^17^ established statistical significance between CNR, CNR/res and X-ray energies. Despite the inclusion of a wider range of X-ray energies such as of 26 keV and 60 keV in Lim’s study, sample numbers were not consistent across all investigated energies, whereas in this study, an even number of samples were assessed for each X-ray energy. Furthermore, Lim et al’s^17^ study used DICOM images with greyscale adjustments to accommodate a combined approach of objective and subjective analysis with incorporation of visual grading analysis following assessment by radiologists. The present study employs the original images without any manipulation of the histograms of reconstructed images (such as background removal) to eliminate possible effects of those adjustments on image quality. The establishment of statistical significance between CNR, CNR/res and X-ray energies in Lim et al^17^ may be explained by greyscale adjustments and unequal sample sizes. Nonetheless, minimal changes to values in CNR and CNR/res were reflected in both studies across all investigated X-ray energy levels.

In the low dose PB-CT images used in this study, the CNR/res for all investigated energies and the Vis for 28-32 keV scans were found to be statistically better than for the standard dose reference AB-CT images. It should be noted that while CNR/res was observed to be independent of energy, there was a clear trade-off between SNR/res and Vis, where increasing the energy increased the former while decreasing the latter. However, previous research has demonstrated that CNR/res and Vis are more relevant to subjective image quality as perceived by human observers.^17^ Overall, it can be argued that 30-32 keV is the optimal X-ray energy range for PB-CT scans using the imaging setup similar to the one in this study.

This study alludes to 30-32 keV being the optimal X-ray energy in PB-CT for an average size and breast density when using synchrotron radiation and flat panel detectors. Discrepancies between different studies can be attributed to different experimental set-ups and detectors used. For example, a phantom-based study by Oliva et al,^15^ utilised CNR as a prime indicator of image quality to investigate the effect of X-ray energy, deducing that lower X-rays energies, in particular 28 keV warrants maximum CNR when using the photon-counting Pixirad-8 image detector. Although flat-panel detectors are widely used in imaging, technological developments have facilitated direct conversion of X-ray photons into electrical charge. This technology is employed in photon-counting detectors to delineate individual photons in each pixel.^24^ This may allow for superior image quality at lower X-ray energies and reduced radiation doses.

On the other hand, Arhatari et al^25^ compared image quality between two flat panel detectors: the Hamamatsu and Xenios detector, indicating that the Hamamatsu detector demonstrated slightly better spatial resolution, whilst the Xenios detector yielded better SNR/res. While the Xenios detector was highly efficient with an increased frame rate, the study concluded that image quality between both detectors were comparable.

The study conducted by Delogu et al^26^ also acknowledged the influence of breast composition on optimal X-ray energy. At a fixed dose, breast thickness and glandularity define the energy needed to maximise CNR. The study concluded that in breasts that are between 8–16 cm in diameter and glandularity of up to 0.25, an X-ray energy of 28 keV can achieve CNR values that are greater than 93% of its potential maximum. This indicates that some differences in the findings of the present study compared to other studies may be attributed to variations in breast composition across the mastectomy samples. While all mastectomy samples used in this study were fitted in the same 11 cm container, breast composition was not measured across the samples. The results from this study may not be replicable in breasts that, for example, are extremely large in size or have increased glandularity as higher X-ray energies are likely to be needed to penetrate through thicker and denser breast tissues.

Preliminary research into optimisation of image quality in PB-CT has been promising, with results demonstrating superior image quality than conventional techniques such as AB-CT at clinically acceptable doses. Nevertheless, image quality optimisation can be separated into a magnitude of other factors that are not limited to pre-processing, detector types, experimental set-ups, and post-processing. One important limitation is the prevalent use of mastectomy samples in current research and the fact that very little is known about how image quality in PB-CT is affected by the presence of adjacent tissues to the breast such pectoral muscles as well as involuntary patient motions. Thus, further research into image optimisation will be required after the initial clinical trials.

## Conclusion

This study was aimed to investigate the effect of X-ray energy and radiation dose on image quality metrics in PB-CT imaging in comparison to AB-CT. Through an objective assessment of image quality by measuring SNR, SNR/res, visibility, CNR and CNR/res, the results indicate that X-ray energy is an important factor that influences SNR and SNR/res significantly and to a lesser degree visibility in PB-CT. This study also demonstrated that 4 mGy PB-CT images had superior image quality (based on all metrics) and 2 mGy PB-CT images showed higher CNR/res and vis compared to 4mGy AB-CT images.
